# Evaluation of Slug Refuge Traps in a Soybean Reduced-Tillage Cover Crop System

**DOI:** 10.3390/insects12010062

**Published:** 2021-01-12

**Authors:** Amy L. Raudenbush, Adrian J. Pekarcik, Van R. Haden, Kelley J. Tilmon

**Affiliations:** 1 Ohio Agricultural Research and Development Center, Department of Entomology, The Ohio State University, 1680 Madison Ave., Wooster, OH 44691, USA; Pekarcik.4@osu.edu (A.J.P.); Tilmon.1@osu.edu (K.J.T.); 2 Agricultural Technical Institute, The Ohio State University, 1328 Dover Rd, Wooster, OH 44691, USA; haden.9@osu.edu

**Keywords:** mollusks, monitoring, integrated pest management, field crops, no-till, shingle trap

## Abstract

**Simple Summary:**

Slugs have become more frequent pests of field crops, including soybean. Monitoring slugs during the day is difficult because slugs are nocturnal, so trapping is often used to monitor populations. A variety of traps have been developed, though there are few direct comparisons of the different trap types. The objective of this study was to compare trapping efficiency of two types of slug refuge traps in soybeans. We tested a traditional shingle trap and a modified shingle trap with a water-filled pitfall trap beneath it. The modified shingle traps captured significantly more slugs than the traditional shingle trap, mainly due to the pitfall component (which was significantly cooler than the shingle component). As slug density decreased, this trend was most pronounced, suggesting that the modified shingle trap is a more sensitive sampling tool which may be useful in agronomic slug research.

**Abstract:**

As more farmers adopt no- or reduced-tillage and/or cover crop land management practices, slugs have become more frequent pests of field crops, including soybean. Monitoring slugs visually is difficult because they are nocturnal, so several trapping methods have been developed, though comparisons of trap types are rare. The objective of this study was to compare trapping efficiency of two types of slug refuge traps in reduced-tillage soybeans following cover crop termination. We tested a traditional shingle trap and a modified shingle trap with a water-filled pitfall trap beneath it. Traps were deployed in 24 pairs in 2018 and 2019 in experimental soybean plots. We counted slug captures weekly over a 5-week time period each year. In 2018, we counted the total number of slugs under each trap type. In 2019, counts were categorized into specific trap components (shingle vs. in/on/under the pitfall). Temperature was also recorded in 2019. The modified shingle traps captured significantly more slugs than the traditional shingle traps, mainly due to the pitfall component. This trend was most pronounced as slug density decreased, suggesting that the modified shingle trap is a more sensitive sampling tool which may be particularly valuable when used for research purposes.

## 1. Introduction

The importance of terrestrial mollusks, primarily slugs, as non-insect pests of agronomic crops, including corn (*Zea mays* L.) and soybean (*Glycine max* L.), has been well documented [1,2,3,4,5,6,7,8]. There are four main slug species that have been reported in field crops in the northeastern U.S., including the gray field slug, *Deroceras reticulatum* (Müller), marsh slug, *D. laeve* (Müller), dusky slug, *Arion subfuscus* (Draparnaud), and banded slug, *Arion fasciatus* (Nilsson) [6,7]. Each species causes similar damage. In soybean, damage is primarily to seedlings and/or young soybean plants [9,10,11]. Spring planting coincides with the cooler temperature and higher humidity favored by slugs, putting seeds, cotyledons, and new foliage at risk. This can cause decreased emergence, plant stand losses, and/or reduced yields [7,9,11]. In rare cases, plant stand loss can be so severe that replanting is needed [4]. Slug damage is a particular problem in systems with reduced tillage intensity (e.g., no-till or conservation tillage) or following cover crops where crop residues and microenvironmental conditions are favorable for slugs [1,2,3,4,5,7,12,13,14]. For instance, Musick found that slugs cause up to 60% plant stand loss in no-till field corn, but only 5% loss in conventional tillage systems [1].

Monitoring pest populations is a critical component to successful decision-making in an integrated pest management (IPM) program [7,15]. However, slugs are difficult to monitor because they are primarily nocturnal [16,17]. Additionally, slug damage can be confused with other pests [4]. Hammond observed soybean defoliation patterns from slugs to be similar to other soybean pests present in the spring, such as the bean leaf beetle, *Cerotoma trifurcata* (Förster) or the Mexican bean beetle, *Epilachna varivestis* Mulsant [4]. Thus, relying solely on foliar damage to diagnose pest problems can lead to misidentification and potentially unnecessary and ineffective insecticide applications.

Since visual monitoring is not practical and damage assessment is unreliable, other slug monitoring methods have been explored, including defined-area traps [18,19,20,21], cold water extraction [20,22], soil searching [20,22], and most commonly, refuge traps [6,7,13,14,16,18,23,24,25,26,27,28]. A refuge trap is a temporary shelter that takes advantage of slug behavior to seek moisture and cover during times of inactivity during the day, which offers scouting convenience [7,25,27]. Examples of refuge traps used to monitor slugs include stones, leaves, tiles [16], turf placed grass side down [23] light-proofed wooden boxes [24], wooden boards [28], and variations of roofing shingles [7,13,25]. Schrim and Byers evaluated six refuge monitoring traps against three common slug species in a sod-seeded legume system [25]. Slugs showed a preference for shingles covered in aluminum foil, which reflected sunlight and reduced temperature under the shingle, creating a cooler shelter [25]. However, the aluminum foil was attractive to wildlife which resulted in occasional destruction of traps.

Refuge traps have also been modified to include space and/or attractants/baits underneath the trap [6,23,26]. Thomas used a metaldehyde bait to attract slugs, however, results were inconsistent due to environmental conditions [23]. These inconsistencies led Thomas to construct a refuge trap using a 15.2 cm square piece of glass painted black to place over the bait. Thomas noted more consistent results once the refuge trap was placed over the metaldehyde bait [23]. Calvin and Losey used a 15.2 cm square roofing shingle covered with aluminum foil with a 10.2 cm × 15.2 cm hole in the soil under the shingle in their trial [26]. However, the objective of the study was to evaluate slug baits in corn rather than to assess the efficacy of the trapping method, and no data are available on the trapping value of the space under the shingle. Hammond et al. also modified an aluminum foil covered shingle trap by placing a cup of beer as an attractant underneath [6]. Despite beer effectively capturing slugs [6], beer is rarely obtainable using university purchasing accounts. Additionally, baits and attractants lose attractiveness over time, requiring additional effort for maintenance and replacement [28,29]. Currently, the monitoring trap recommended for use in the mid-Atlantic region, is a 30.5 cm × 30.5 cm white roofing shingle [7]. White was chosen to reduce heat under the trap.

Despite the use of several trapping methods to monitor slug populations, there is little research comparing the attractiveness of these trap types to slugs in reduced-tillage cover crop soybean systems. The objective of this study was to compare two different refuge trap designs: a traditional refuge white shingle trap and a modified shingle trap with the addition of a water-filled pitfall trap underneath the shingle. We hypothesized that more slugs would be sampled from the modified trap than the traditional trap because of a cooler and moister microenvironment from the pitfall trap component.

## 2. Materials and Methods

Two types of slug refuge traps were evaluated as paired samples in reduced-tillage cover crop soybean fields. Slugs were counted weekly from 18 June to 16 July 2018 and again from 20 June to 17 July 2019 at the King and Fry Farms, respectively, located at the Ohio Agricultural Research and Development Center (Wooster, OH, USA). This timeframe was chosen because it is when slugs were most abundant in the soybean field. Both years, the fields were previously planted as corn and residue was present throughout the slug monitoring period. After the corn was harvested, a cover crop was established in the autumn of 2017 and 2018 at each location. Cover crops and plot size varied in the two years. In 2018, soybean was planted into various cover crops in 3.0 m × 9.1 m plots containing one of 15 cover crop treatments and replicated four times in a randomized complete block design. For the trap study, only six cover crop treatments were used; crimson clover, red clover, annual ryegrass, tillage radish, forage collard, and a no cover crop control, for a total of 24 paired traps located in 24 plots. In 2019, soybean was planted into 9.1 m × 9.1 m plots of cereal rye that were terminated at three different time periods, plus a no cover crop control, replicated six times in a randomized complete block design. All plots were utilized, for a total of 24 paired traps located in 24 plots.

### 2.1. Assembly of the Traditional and Modified Refuge Traps

**Figure 1:**
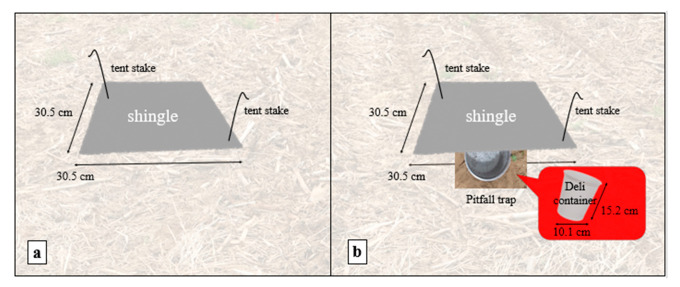
(**a**) Traditional shingle trap and (**b**) modified trap with the shingle placed over a pitfall trap.

**Figure 2:**
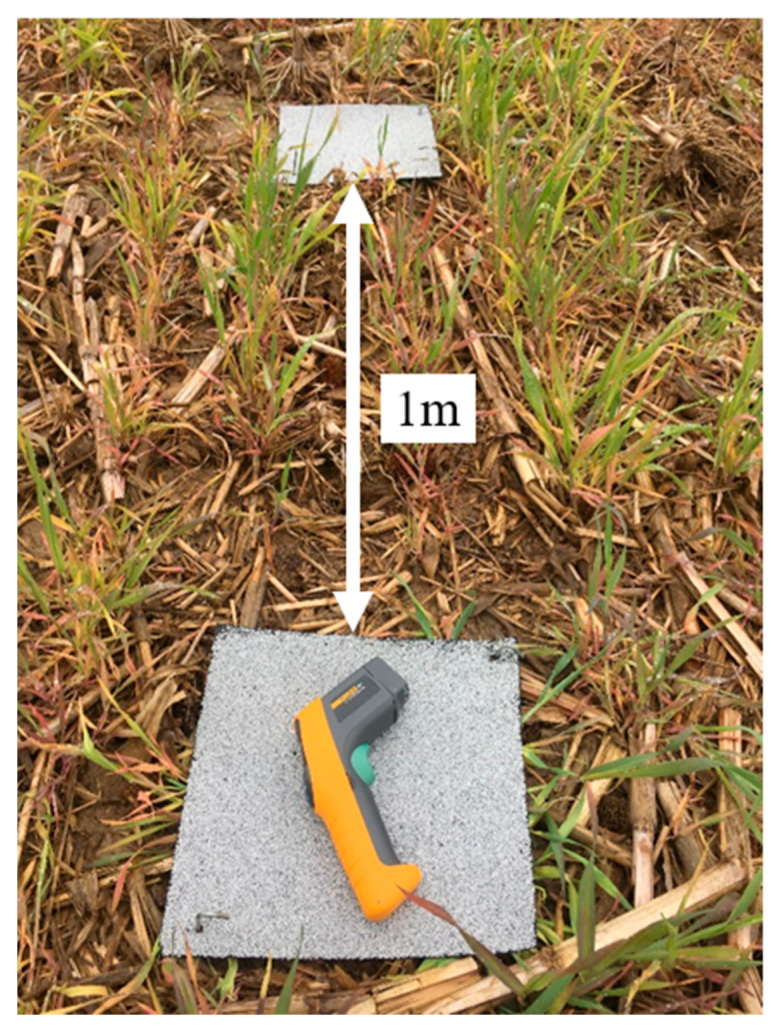
Paired traditional and modified refuge traps in cereal rye cover crop, 2019, and the infrared thermometer used for measuring temperature.

The traditional trap consisted of a 30.5 cm × 30.5 cm Shasta white roofing shingle (Owens Corning^®^, Toledo, OH, USA), after Douglas and Tooker [3]. Shingles were placed directly over residue and secured with two galvanized tent stakes in opposing corners (ALAZCO^™^, Stilwell, KS, USA) (Figure 1a). The modified trap consisted of a 30.5 cm × 30.5 cm Shasta white shingle with a pitfall trap underneath. The pitfall was a 0.95 L white deli container filled ½ way with water to which a drop of dish detergent had been added (to reduce surface tension). The pitfall trap was emptied and refilled with soapy water weekly. The container was placed in a hole made by a golf hole cup cutter so the rim was flush with the soil surface (Figure 1b). The shingle was placed over the pitfall and secured as described above. The traditional and modified refuge traps were deployed as pairs, set 1 m apart from each another in each plot (Figure 2).

### 2.2. Data Collection

In both 2018 (King Farm) and 2019 (Fry Farm), the traps were checked for the presence of slugs weekly for five consecutive weeks beginning the week of 17 June. In 2018, for the traditional trap, all slugs (juveniles and adults) found under each shingle were recorded. For the modified trap, the number of slugs per trap (under the shingle and in, on or under the pitfall) were recorded as a total. In 2019, traditional traps were counted similar to 2018; however, for the modified slug traps, numbers were recorded separately for slug location (under the shingle component or in, on, or under the pitfall component). Throughout the data collection, all live slugs were left undisturbed. Slugs found in the pitfall trap were dead and discarded weekly when new soapy water was added to the trap. We did not identify slugs to species due to time and resource limitations; however, previous research found the most common species in Ohio field crops to be *D. reticulatum* and *D. laeve* [6]. In addition, in 2019, we also recorded the surface temperature under the traditional trap shingle, and under each component of the modified trap (shingle and pitfall), at the time of slug sampling, using an infrared thermometer (FLUKE-63; Fluke Corporation, Everett, WA) (Figure 2). To minimize diurnal variability in temperature and slug sheltering behavior, all field evaluations began approximately between 9:00–10:00 a.m. Evaluations were completed within an hour from the starting time.

In addition to weekly slug counts, weather data were also taken daily throughout the experiment from a solar powered Campbell Scientific datalogger and modem located on the Ohio Agricultural Research and Development campus in Wooster, Ohio (USA). These data were used to record precipitation for the duration of the experiment in 2018 and 2019.

### 2.3. Statistical Analysis

For each site, the average number of slugs sampled from, and temperature recorded for each trap type per paired sample across all sampling dates were analyzed separately for normality using PROC UNIVARIATE [30]. Temperature data were normal and used without transformation in subsequent analyses. Slug data were non-normal (*p* < 0.05) for both trap types and transformed using log(x + 1) prior to further analysis. Both datasets were also assessed for homogeneity and homoscedasticity with Levene’s tests using PROC GLM [30]. Slug data at King Farm violated norms of homogeneity and homoscedasticity (*p* < 0.05).

It is important to note that this trap experiment was performed against the background of different experiments (two cover crop studies) with their own experimental design. This required us to perform preliminary analyses to determine the appropriate statistical model. First, preliminary analyses were conducted to determine whether the independent variables site-year, block, and cover crop treatment were significant predictors of the total number of slugs sampled per plot, to guide subsequent analyses. The total number of slugs captured per plot [i.e., from both traditional and modified traps] was averaged across all sampling dates and compared among site-years using PROC MIXED [30], with multiple comparisons performed using Tukey’s post-hoc test with LSmeans ≤ 0.05. For King Farm data only, a Satterthwaite approximation for the degrees of freedom was used to account for unequal variances. The average number of slugs significantly varied among site-year (F = 55.92, df = 1, 46, *p* < 0.0001). Subsequent analyses were performed separately by site-year to assess whether block and cover crop treatment were significant predictors of the average number of slugs sampled per plot across all sampling dates using PROC MIXED with multiple comparisons performed using Tukey’s post-hoc test with LSmeans ≤ 0.05. The average number of slugs per plot did not significantly vary among block (King Farm: F = 0.45, df = 3, 15, *p* = 0.7205; Fry Farm: F = 1.55, df = 5, 15, *p* = 0.2344) or cover-crop treatment (King Farm: F = 0.65, df = 5, 15, *p* = 0.6685; Fry Farm: F = 1.25, df = 5, 15, *p* = 0.3275). As a result, all subsequent analyses were performed separately by site-year, and with block and cover crop treatment designated as random effects in repeated measures and regression analyses.

The average numbers of slugs sampled from the traditional and modified traps of each plot were compared over time in separate analyses by site-year [i.e., King Farm 2018 or Fry Farm 2019] with respect to sampling date, trap type, and sampling date*trap type interaction using repeated measures ANOVA with PROC MIXED [30]. Block and cover crop treatment were designated as random effects and multiple comparisons were performed separately for each sampling date using Tukey’s post-hoc test with LSmeans ≤ 0.05. The rates at which the traditional and modified traps sampled slugs within the same plot were compared for King Farm and Fry Farm in separate linear regression analyses using PROC REG [30]. First, the data sets were evaluated for the presence of influential outliers with robust linear regression using PROC ROBUSTREG [30]. No influential datapoints were detected for King Farm, so regular linear regression was performed; however, four were identified at Fry Farm, so robust regression was used to better account for the outliers while estimating the slope.

For 2019 Fry Farm data, the total number of slugs sampled, and the average temperature recorded from under the different trap components (i.e., traditional trap shingle, modified trap shingle, and modified trap pitfall) for each paired sample were compared with respect to sampling date, trap component, and sampling date*trap component interaction in separate analyses with a nested repeated measures ANOVA using PROC MIXED. Trap component was nested within trap type for the analysis and block and cover crop treatment were designated as random effects. Multiple comparisons were performed separately for each evaluation date using Tukey’s post-hoc test with LSmeans ≤ 0.05.

## 3. Results

### 3.1. Slug Density

**Figure 3:**
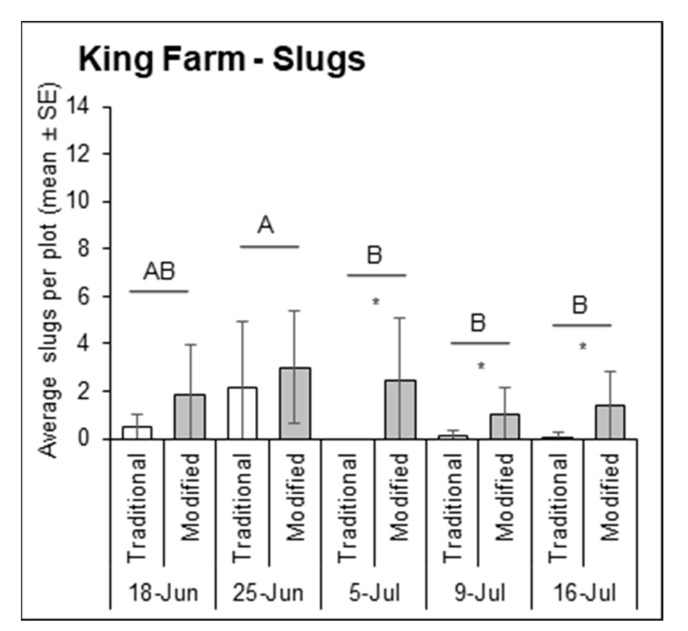
Average number of slugs sampled from each traditional trap (white bars) and modified trap (gray bars) for each sampling date in 2018 at King Farm in Wooster, OH, USA. Uppercase letters represent significant differences (*p* < 0.05) from multiple comparisons analyses comparing total slugs per plot over time. Asterisks above bars indicate significant differences (*p* < 0.05) in the number of slugs caught by the traditional and modified traps for that sampling date.

**Figure 4:**
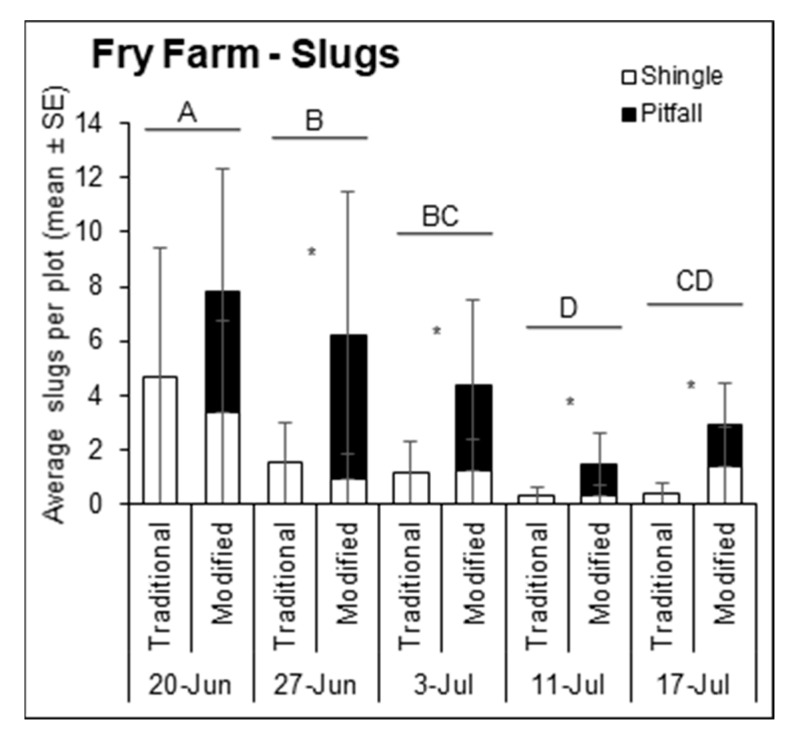
Average number of slugs sampled from under the traditional (left bar) and modified (right bar) traps for each sampling date in 2019 at Fry Farm in Wooster, OH, USA. Data from the modified trap are broken down into components (shingle, white vs. pitfall, black). Uppercase letters represent significant differences (*p* < 0.05) from multiple comparisons analyses comparing total slugs per plot over time. Asterisks indicate significant differences (*p* < 0.05) in the number of slugs caught by the traditional and modified traps for that sampling date.

At King Farm in 2018, the number of slugs sampled differed significantly by sampling date (Figure 3; F = 4.83, df = 4, 143, *p* = 0.0011). Of five sampling dates, the last three had lower slug density. The initial slug population sampled on 18 June (1.2 ± 1.6) was similar to the peak population on 25 June (2.6 ± 2.5), after which populations significantly declined. At Fry Farm in 2019, slug densities also varied by sampling date (Figure 4; F = 38.13, df = 4, 222, *p* < 0.0001), and the initial population sampled on 20 June (6.3 ± 5.3) was significantly greater than on all subsequent sampling dates, which declined through 11 July (0.8 ± 1.4) after which they did not recover.

### 3.2. Slug Captures

At King Farm in 2018, the traditional and modified traps caught significantly different numbers of slugs overall (Figure 3; F = 43.80, df = 1, 135, *p* < 0.0001) but not for the trap*time interaction (F = 1.79, df = 4, 135, *p* = 0.1334). Nearly 8 times as many slugs were sampled on average in the modified traps (1.8 ± 2.0) compared to the traditional traps (0.2 ± 0.9) (t = 4.82, df = 150, *p* < 0.0001). Both trap types caught similar numbers of slugs on 18 June and 25 June when slug populations were high *(p* > 0.05), however, the modified trap caught significantly more slugs than the traditional trap on the last three sampling dates including 5 July (*t* = 3.37, df = 222, *p* = 0.0009), 9 July (t = 2.93, df = 222, *p* = 0.0037), and 16 July (*t* = 4.52, df = 222, *p* < 0.0001), when there were fewer slugs. On Fry Farm in 2019, the number of slugs caught by the traditional and modified traps significantly varied overall (F = 38.13, df = 4, 222, *p* < 0.0001) but not over time (F = 1.54, df = 4, 222, *p* = 0.1907). The modified traps caught significantly more slugs on four out of the five sampling dates (Figure 4; F = 34.69, df = 1, 222, *p* < 0.0001), each during the period of lower slug density after the initial peak on 20 June. The modified trap caught significantly more slugs than the traditional trap on 27 June (*t* = 9.38, df = 222, *p* < 0.0001), 3 July (*t* = −3.37, df = 222, *p* = 0.0298), 11 July (*t* = 3.61, df = 150, *p* = 0.0004), and 17 July (*t* = −4.52, df = 222, *p* = 0.0004). There was no significant correlation between the traditional and modified traps for slug capture rates at either site-year (King-2018 linear regression: R2 = 0.11, F = 2.80, df = 1, 22, *p* = 0.1086; Fry-2019 robust regression: R2 = 0.01, X2(1, 22) = 2861.20, *p* = 0.2741).

### 3.3. Slug Captures by Trap Component

On Fry Farm in 2019, in the modified traps, we separately counted the number of slugs associated with the shingle portion of the trap, and in, on, and under the pitfall portion of the trap (Figure 4). Across all sampling dates, the pitfall component captured nearly twice as many slugs (3.1 ± 4.8 slugs) as the shingle component (1.5 ± 2.0 slugs) of the modified traps (F = 19.90, df = 1, 320, *p* < 0.0001). Multiple comparisons tests indicated that slug captures from the shingle component of the modified trap did not differ from captures from the traditional shingle trap (1.6 ± 2.4) (*p* > 0.05) on any sampling date. Significant differences in the number of slugs sampled per trap type and component were also detected over time; Trap component (pitfall/shingle; F = 5.52, df = 4, 320, *p* = 0.0003) rather than trap type (traditional/modified; F = 2.48, df = 4, 320, *p* = 0.0437) drove this pattern.

### 3.4. Temperature by Trap Component

**Figure 5:**
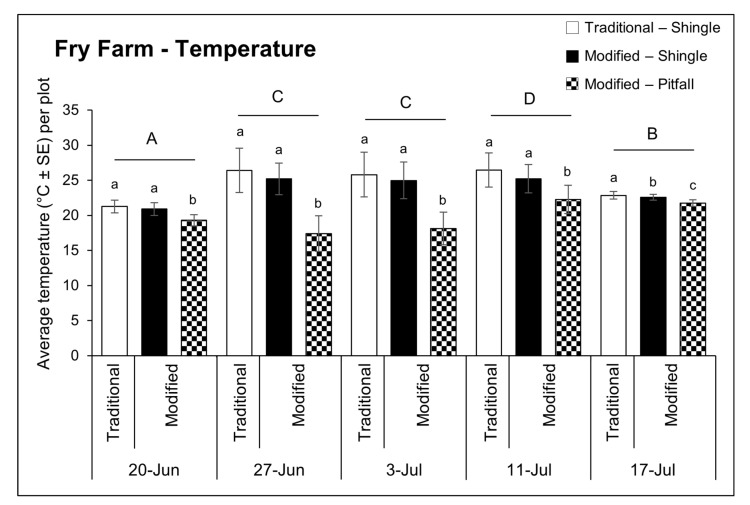
Average temperature recorded under each trap component including the traditional trap shingle (white bars), modified trap shingle (black bars) and modified trap pitfall trap (black and white checkered bars) for each sampling date in 2019, at Fry Farm in Wooster, OH, USA. Uppercase letters represent significant differences (*p* < 0.05) from multiple comparisons analyses comparing the average temperature for all traps and components per plot over time. Lowercase letters indicate significant differences (*p* < 0.05) in the number of slugs found under each trap component for each sampling date.

The average temperature recorded under each trap type (F = 17.99, df = 4, 320, *p* < 0.0001) and component (F = 36.67, df = 4, 320, *p* < 0.0001) significantly varied over time (Figure 5). Average trap temperatures were significantly warmer on 11 July (24.5 ± 2.8 °C) than any other sampling date and the fewest slugs per trap were recorded at this time (F = 53.42, df = 4, 320, *p* < 0.0001). There was a significant relationship between trap type and temperature (F = 208.37, df = 1, 320, *p* < 0.0001); modified traps (21.7 ± 3.3 °C) were approximately 3 °C cooler on average than traditional traps (24.6 ± 3.1 °C). Temperature also differed under each trap component (F = 322.80, df = 1, 320, *p* < 0.0001). The pitfall portion of the modified traps was on average cooler (19.7 ± 2.7 °C) than the temperature under the shingle component of the modified trap (23.8 ± 2.5 °C) (*t* = −17.97, df = 320, *p* < 0.0001) or under the traditional shingle trap (24.6 ± 3.1 °C) (*t* = −21.48, df = 320, *p* < 0.0001)—a pattern which did not vary by sampling date. Temperature under modified trap and traditional trap shingles was similar on all sampling dates except for 17 July (*t* = −2.63, df = 45, *p* = 0.0309).

## 4. Discussion

The increasing popularity of no- or reduced-tillage and cover crop systems has led to increased slug problems for such farmers, which puts a greater premium on good slug scouting techniques in IPM and slug research programs. Shingle refuge traps have come into common use for these purposes [7,25] because they are inexpensive and convenient, but variation in the sampling efficacy among different versions of the shingle trap has not been well-studied. This study compares a traditional shingle trap with a modified trap featuring a shingle placed over a water-filled pitfall trap. The modified trap design was based on our observations in other studies that insect pitfall traps tended to collect slugs, perhaps because of cooler and moister conditions which are known to attract them.

Slug populations are known to vary from year to year [6]. The average slug population in our study was approximately three times higher in 2019 than 2018, perhaps because of higher rainfall in 2019 (47.2 cm from May–July 2019, vs. 31.5 cm from May–July 2018), or perhaps because the location differed each year. This allowed the monitoring methods to be evaluated under different population pressures. Nonetheless, similar patterns were seen in both site-years. Slug populations generally decreased over time from June to July, which is consistent with typical patterns of agricultural damage which tend to decrease as summer advances [7].

In both site-years, the modified trap sampled more slugs on average—a trend which was most pronounced when slug populations were lower. Broken down by trap components (shingle vs. pitfall) in 2019, slug captures did not vary with either type of trap shingle, but increased with the pitfall component of the trap, which remained significantly cooler than the shingles on every sampling date. This is consistent with previous findings that showed that *D. reticulatum*, the gray field slug prefers traps with a temperature of 17.0–18.0 °C [27] and will migrate to a more favorable microenvironment when the temperature under a refuge trap gets too high. Slug captures decreased significantly with increasing trap temperature in our study, and showed a trend to increase in, on, and/or under the pitfall component as numbers decreased in the shingle component. Similar to the use of beer traps evaluated by Scaccini et al. [28], the water-filled pitfall component of the modified trap captures slugs permanently (accumulated over 7 days), whereas slugs are free to leave the shingle portion of the trap. This could also have contributed to the higher number of slugs we found associated with the pitfalls. However, many slugs were also counted alive and well underneath the pitfall container. We counted slugs in/on/under the pitfalls but did not separate these counts, so we are unable to assess the proportion permanently captured/accumulated, vs. those able to move on.

It is relevant to note that there was no significant correlation between slug captures by the traditional vs. modified traps in either site-year. Stated another way, these two types of traps did not tell the same story about the underlying slug populations in the sampling locations. The modified trap was shown to capture more slugs relative to the traditional trap in particular when slug populations were lower. Thus, this method is probably the more sensitive sampling tool especially at lower densities. This might matter more in a research context than in a pest management context, where the goals differ (i.e., precision population data vs. relative pest abundance). We did not record the time required to check each type of trap but can anecdotally assert that it took longer to process (and maintain) the modified traps on a weekly basis. The modified shingle trap could prove to be a superior sampling tool for slugs than traditional shingles in agricultural research, but the greater time investment might make it less practical for IPM monitoring. Future work could evaluate the cost-benefit of these sampling methods more specifically in an IPM context.

## 5. Conclusions

Refuge traps are convenient and popular methods for sampling slug populations. A modified shingle trap with a pitfall trap underneath captured more slugs than a shingle trap alone, due to greater captures in the pitfall component, which also remained cooler than the shingles. This difference was more pronounced at lower slug densities, suggesting that the modified shingle trap is a more sensitive sampling tool in particular for research purposes, where sampling sensitivity may be prized over speed of trap monitoring.

## Data Availability

The datasets used and/or analyzed in this study are available upon agreement with the corresponding author.
